# Do 6-Month Motor Skills Have Cascading Effects on 12-Month Motor and Cognitive Development in Extremely Preterm and Full-Term Infants?

**DOI:** 10.3389/fpsyg.2020.01297

**Published:** 2020-06-26

**Authors:** Mariagrazia Zuccarini, Annalisa Guarini, Silvia Savini, Giacomo Faldella, Alessandra Sansavini

**Affiliations:** ^1^Department of Psychology, University of Bologna, Bologna, Italy; ^2^Department of Medical and Surgical Sciences, University of Bologna, Bologna, Italy

**Keywords:** gross motor skills, motor development, cognitive development, extremely preterm infants, first year of life

## Abstract

In this study, we analyzed whether 6-month gross and fine motor skills were related to 12-month gross and fine motor skills and cognitive development, controlling for 6-month cognitive skills, and neonatal status (extremely low gestational age ELGA ≤ 28 weeks vs. full-term FT ≥ 37 weeks). We also investigated, at 6 months, predictive indexes for motor and cognitive delay at 12-months. We assessed 40 infants (20 ELGA and 20 FT) at 6 and 12 months (corrected age for the ELGA infants). Six-month gross motor skills were related to 12-month gross motor, fine motor, and cognitive skills and predicted 12-month gross motor delay. Six-month cognitive skills explained an additional amount of variance of 12-month gross motor skills, whereas neonatal status explained an additional amount of variance of 12-month cognitive skills. Considering the intradomain and cross-domain cascading effects of early gross motor skills on later motor and cognitive development, these skills should be repeatedly assessed in ELGA infants in the first year of life for early identification of infants with delayed gross motor skills and implementation of customized interventions.

## Introduction

The survival rate of extremely low gestational age (ELGA) infants, that is, with a gestational age ≤ 28 weeks, has considerably improved over the last 20 years (Ruegger et al., 2012; Johnson and Marlow, 2017). Nevertheless, these infants have a high risk for developmental delays across multiple domains, even in the absence of major cerebral damage (de Kievet et al., 2009; Mansson and Stjernqvist, 2014; Sansavini et al., 2014; Lefebvre et al., 2016). Motor development is particularly affected in ELGA infants during the first months of life and throughout childhood and adolescence (de Kievet et al., 2009; Sansavini et al., 2011, 2014; Mansson and Stjernqvist, 2014; Lefebvre et al., 2016; Fuentefria et al., 2017). de Kievet et al. (2009) reported in their meta-analysis that preterm children with a mean gestational age of 28.2 weeks are on average -0.57 to -0.88 SD behind their full-term (FT) peers (gestational age ≥ 37 weeks) in motor development from the first years of life to 15 years of age.

Concerning gross motor skills, some studies have reported that ELGA infants lagged behind FT peers from the first months of life up to 24 months (Sansavini et al., 2011; Yaari et al., 2018). The gap between ELGA and FT infants in gross motor development persisted (Yaari et al., 2018) and increased up to the third year of life, as shown by a further study (Sansavini et al., 2014). Other studies have examined specific aspects of gross motor functioning in the ELGA population, pointing out atypical trajectories in the acquisition of motor milestones in extremely preterm infants throughout the first 18 months of age (Pin et al., 2009, 2010). Indeed, between 4 and 8 months, these infants showed an uneven progression of the sitting posture, and at 8 months of age, only 56% of the ELGA infants had achieved a stable unsupported sitting posture compared to 90% of the FT infants (Pin et al., 2009). The gap in gross motor development between ELGA and FT infants persisted up to 18 months of age, because at this age, one third of infants in the preterm sample had not yet reached mature mobility and independent walking (Pin et al., 2010). In addition, a few studies examined the prevalence of moderate-severe delays (scores below 2 SD) in gross motor development in the first years of life. At 24 months, 16.6% of the extremely preterm children, assessed by the EPIPAGE-2 study through a parental questionnaire, scored below 2 SD with respect to the normative values (Pierrat et al., 2017); at 30 months, 7% of the extremely preterm children, assessed by the EXPRESS study through a standardized instrument, scored 2 SD below the mean of their control group (Mansson and Stjernqvist, 2014).

Concerning fine motor skills, evidence in studies using standardized assessments revealed that ELGA infants obtained lower eye-hand coordination scores from 1 to 24 months of age than their FT peers, with a significantly increasing gap over time (Sansavini et al., 2011; Yaari et al., 2018). In another study, researchers reported that ELGA infants received lower fine motor scores than FT infants at 12, 24, and 30 months (Sansavini et al., 2014). Focusing on object exploration it was found that, compared to controls, ELGA infants explored objects for a shorter duration (Lobo et al., 2015), showed less advanced oral and manual exploratory behaviors at 6 months (Zuccarini et al., 2016), and uneven developmental exploratory patterns between 6 and 9 months than their FT peers (Zuccarini et al., 2017). Concerning the prevalence of moderate-severe delays (scores below 2 SD) in fine motor development in the first years of life, the EXPRESS study found that 12.4% of the extremely preterm children at 30 months, assessed through a standardized instrument, scored 2 SD below the mean of their control group (Mansson and Stjernqvist, 2014).

Together, these findings highlight that extremely preterm birth is associated with poor motor skills and suggest the relevance of assessing the motor development of ELGA infants in follow-up programs. This is crucial for identifying which motor behaviors can predict later motor development (Evensen et al., 2009; Charitou et al., 2010) and for differentiating preterm infants at higher risk of developing motor impairments in order to facilitate targeted interventions as early as possible (Zwicker, 2014; Lefebvre et al., 2016).

Despite this evidence, studies examining the predictive value of early motor assessment on later motor development in the ELGA population are very scarce. In the study by Lefebvre et al. (2016), gross motor scores at 4, 10, and 12 months predicted gross motor scores at 18 months. Specifically, ELGA infants not delayed in gross motor development across the first year of life obtained higher scores at 18 months. However, that study investigated the associations between early and later motor skills without considering the interrelations among motor subdomains. In addition, it included ELGA infants with neurological damage and did not include a control group.

Early motor development can also have cascading effects on other developmental domains. A recent growing body of evidence has revealed that early motor milestones are strictly associated with cognitive functions in FT infants as well as in ELGA infants (Lefebvre et al., 2016; Oudgenoeg-Paz et al., 2017; Zuccarini et al., 2017). As shown in the review by Oudgenoeg-Paz et al. (2017), specific gross motor behaviors such as early postural control or general spontaneous movements in the first months of life are predictive of cognitive skills assessed by the first year of life and during childhood in preterm children. For example, Lefebvre et al. (2016) found that gross motor scores, across the first year, predicted, not only motor scores, but also cognitive scores at 18 months in ELGA infants with neurological damage. However, Lefebvre et al. did not control (a) for early cognitive development that could partially explain the relationship between the motor and cognitive domains and (b) the specific contribution of early gross and fine motor skills to 12-month cognitive development in the ELGA population.

We designed this study with three main objectives. First, we examined whether gross and fine motor skills at 6 months were related to gross and fine motor skills at 12 months of age, controlling for 6-month cognitive performance and neonatal status (ELGA vs. FT status). Based on previous studies (Lefebvre et al., 2016), we expected to find significant intra-domain relationships. Second, we examined whether gross and fine motor skills at 6 months were related to cognitive skills at 12 months, controlling for 6-month cognitive performance, and neonatal status. Based on previous studies, we expected to find significant cross-domain relationships; in particular, we expected that gross motor skills (Lefebvre et al., 2016; Oudgenoeg-Paz et al., 2017) will predict later cognitive development. Finally, we explored whether gross and fine motor skills at 6 months predicted a delay in fine and gross motor development and in cognitive development at 12 months, controlling for 6-month cognitive performance and neonatal status. Based on previous studies (Lefebvre et al., 2016), we expected that early motor skills, in particular poor gross motor skills, will predict a subsequent motor and cognitive delay.

## Materials and Methods

### Participants

The sample included 40 Italian monolingual infants, 20 ELGA and 20 FT, all living in Emilia-Romagna, a region in Northeast Italy. The ELGA infants were born at the Neonatal Intensive Care Unit of Bologna University hospital; the FT infants were recruited at the same hospital. All infants had no major cerebral damage, congenital malformations, or visual or hearing impairments. The ELGA and FT infants’ biological, medical, and sociodemographic characteristics are described in Table 1. The two groups were comparable in gender, maternal education level, and maternal age.

**TABLE 1:** Biological, socio-demographic, and medical characteristics of participants.

	**ELGA (*n* = 20)**	**FT (*n* = 20)**
GA, mean (SD), range (weeks)	25.7 (1.5) 23.2–28.5	39.6 (1.2) 38–42
BW, mean (SD), range (grams)	803 (191) 509–1093	3476 (464) 2430–4200
Male, *n* (%)	9 (45)	11 (55)
First born, *n* (%)	15 (75)	18 (90)
Mothers with a middle/low educational level ≤ 13 year, *n* (%)	12 (60)	8 (40)
Mothers with a high educational level > 13 year, *n* (%)	8 (40)	12 (60)
Maternal age, mean (SD), range (years)	36.2 (4.8) 27–44	34.6 (3.1) 30–41
Cesarean section, *n* (%)	17 (85)	2 (10)
Multiple births, *n* (%)	6 (30)	0 (0)
BW < 1000 *g*, *n* (%)	16 (80)	0 (0)
MV, *n* (%)	11 (55)	0 (0)
SGA, *n* (%)	2 (10)	0 (0)
RDS, *n* (%)	20 (100)	0 (0)
Apnoea, *n* (%)	6 (30)	0 (0)
BPD, *n* (%)	12 (60)	0 (0)
IVH I/II, *n* (%)	1 (5)	0 (0)
HE (≥14 day), *n* (%)	17 (85)	0 (0)
ROP I/II, *n* (%)	13 (65)	0 (0)
Sepsis, *n* (%)	6 (30)	0 (0)
Hyperbil. with phototer., *n* (%)	16 (80)	0 (0)

*GA, gestational age; BW, birthweight; MV, mechanical ventilation. Medical complications (infants could have one or more medical complication): SGA: infants with a birthweight <10th percentile for gestational age; RDS: respiratory distress syndrome, acute illness coming on within 4–6 h of delivery, characterized clinically by respiratory rate ≥ 60/min, dysponea and respiratory distress; Apnoea: significant apnoea was defined as more than four episodes of apnoea/hour or more than two episodes of apnoea/hour if ventilation with bag and mask was required; BPD: bronchopulmonary dysplasia, need of both supplemental oxygen for ≥28 days and at 36 weeks of post-conceptional age; IVH I/II: intra-ventricular hemorrhage originating within the subependymal germinal matrix filling less than, respectively, 10% (I grade) and 50% (II grade) of the ventricular area on parasagittal view; HE: hyperecogenicity, a prolonged flare ≥14 days without cystic lesions, and/or ventricular dilatation and that resolved completely without any abnormality in its place; ROP I/II: retinopathy of prematurity, vasoproliferative retinopathy that resolved without a specific therapy before the presume date of birth; Sepsis: presence of a positive blood culture and/or clinical and laboratoristic signs; Hyperbil. with phototer.: hyperbilirubinemia needing phototherapy according to the criteria proposed by Gomella (2009).*

### Procedure

This study is part of a longitudinal study that followed the development of ELGA infants from birth to preschool age. For this paper, we considered data on gross motor, fine motor and cognitive skills at 6 and 12 months. The ELGA infants’ age was corrected to take into account their level of neuropsychological maturation, as in many studies conducted on preterm infants in the first 2 years of life (Johnson and Marlow, 2006; Sansavini et al., 2011). At the 6-month assessment, the ELGA infants had a mean corrected age of 6 months and 3 days (SD = 7 days); the FT infants had a mean chronological age of 6 months and 5 days (SD = 12 days). At the 12-month assessment, the ELGA infants had a mean corrected age of 12 months and 6 days (SD = 9 days); the FT infants had a mean chronological age of 12 months and 3 days (SD = 9 days). At both assessments, no significant difference was found between the ELGA infants’ corrected age and the FT infants’ chronological age.

At 6 and 12 months, all infants were administered the revised Griffiths Mental Development Scales 0–2 years (GMDS-R, Griffiths, 1996) by a trained psychologist in a quiet room of the Unit of Neonatology of the Bologna University hospital.

The study met ethical guidelines for human subject protections, including adherence to the legal requirements of Italy, and received formal approval from the local Ethical Committee. All parents of the ELGA and FT infants gave informed written consent for study participation, data analysis, and data publication.

### Materials

Concerning the GMDS-R (Griffiths, 1996), for the current study, the locomotor (gross motor skills), eye and hand coordination (fine motor skills), and performance (cognitive skills) subscales were considered at 6 and 12 months.

The locomotor subscale assesses gross motor skills such as postural control balance and gross body coordination; the eye and hand coordination subscale assesses fine motor skills, such as manual dexterity, and manipulative skills (e.g., visual tracking, reaching and grasping, and object manipulation); the performance subscale assesses cognitive skills, such as planning, completing intentional actions, and representing objects. We calculated the subscale scores by referring to the English normative values, as done in previous studies (Sansavini et al., 2011; Zuccarini et al., 2016, 2017) because an Italian standardization of this scale is not available yet. These scales have been used for clinical and research purposes in follow-up studies of preterm infants in several European countries (see a recent review by Pascal et al., 2018). Satisfactory reliability and validity were reported by the author (Griffiths, 1996), with internal consistency coefficients (using the split-half method) ranging from 0.91 to 0.97 and test–retest reliability ranging from 0.40 to 0.89 for the locomotor subscale, from 0.66 to 0.69 for the eye and hand coordination subscale, and from 0.10 to 0.45 for the performance subscale, becoming higher in the second half of the first year.

According to Griffiths’s (1996) manual, a delay in the locomotor (gross motor), eye and hand coordination (fine motor), and performance (cognitive) subscales was defined as a standardized score lower than 2 SD below the mean (i.e., locomotor and eye and hand coordination: *M* = 100.2, SD = 15.9, and delay score < 68.4; performance: *M* = 100.4, SD = 16.0, and delay score < 68.4). The cut-off of 2 SD below the mean has also been used as a reference for identifying children with moderate-severe delays in gross motor and fine motor skills in the first years of life by some cohort studies, i.e., the EPIPAGE-2 (Pierrat et al., 2017), and the EXPRESS study (Mansson and Stjernqvist, 2014), describing neurodevelopmental outcomes of extremely preterm children.

Along these lines, in the current study, we have found that at 12 months, five (25%) ELGA infants and two (10%) FT infants were delayed in the gross motor domain, one (5%) ELGA infant was delayed in the fine motor domain, and one (5%) ELGA infant was delayed in the cognitive domain. The Fisher exact test did not reveal significant differences between the percentages of ELGA and FT infants with a delay in gross motor, fine motor, and cognitive development at 12 months.

### Statistical Analyses

We ran statistical analyses with SPSS 21.0 for Windows with the significance level set at 5%. We checked data for violation of assumption of normal distribution using the Kolmogorov–Smirnov test.

Preliminary, we conducted descriptive analyses and ANOVAs to ascertain whether differences in gross and fine motor and cognitive scores at 6 and 12 months emerged in function of neonatal status (ELGA vs. FT; see Table 2). Statistical comparisons revealed that the ELGA infants had lower scores than the FT infants on gross motor, fine motor, and cognitive subscales at 6 and 12 months (see Table 2).

**TABLE 2:** Means, Standard Deviations and One-Way Analyses in 6 and 12-month gross motor, fine motor, and cognitive scores.

	**ELGA (*n* = 20)**			**FT (*n* = 20)**			**ANOVA**		
	**Mean**	**SD**	**Range**	**Mean**	**SD**	**Range**	***F***	***p***	***η${}_{p}^{2}$***
**6 months**									
Gross motor	98.7	17.6	57–150	114.2	11.5	97–142	10.83	**0.002**	0.022
Fine motor	97.5	16.6	63–123	109.9	12.4	88–128	7.24	**0.011**	0.016
Cognitive	93.1	18.0	64–117	108.0	12.0	72–126	9.51	**0.004**	0.200
**12 months**									
Gross motor	84.0	20.1	50–117	97.3	17.2	57–125	5.08	**0.030**	0.118
Fine motor	95.7	18.0	63–138	111.2	11.3	95–128	10.60	**0.002**	0.218
Cognitive	92.2	12.5	68–118	109.8	13.4	93–148	18.61	**<0.001**	0.329

*Standardized scores of gross motor (locomotor subscale), fine motor (eye and hand coordination subscale), and cognitive skills (performance subscale) of the Griffiths Mental Development Scales were used. Significant results (*p* < 0.05) are in bold.*

We also ran Pearson correlation analyses to explore relationships between motor and cognitive skills at 6 and 12 months in the whole sample (*n* = 40). Results revealed significant intra-domain and cross-domain relationships among gross motor, fine motor, and cognitive raw scores at 6 and 12 months (see Table 3 for correlations between 6 and 12 months; see Supplementary Tables S1, S2 for concurrent correlations at 6 and 12 months).

**TABLE 3:** Pearson’s correlations among gross motor, fine motor and cognitive scores at 6 and 12 months.

	**12 months**					
	**Gross motor**		**Fine motor**		**Cognitive**	
	***r***	***p***	***r***	***p***	***r***	***p***
**6 months**						
Gross motor	0.727	**<0.001**	0.579	**<0.001**	0.648	**<0.001**
Fine motor	0.508	**0.001**	0.502	**0.001**	0.339	**0.032**
Cognitive	0.663	**<0.001**	0.543	**<0.001**	0.446	**0.004**

*Number of participants = 40. Raw scores of gross motor (locomotor subscale), fine motor (eye and hand coordination subscale), and cognitive skills (performance subscale) of the Griffiths Mental Development Scales were used. Significant results (*p* < 0.05) are in bold.*

Based on these preliminary analyses, we performed hierarchical linear regressions to examine relationships between gross motor and fine motor scores at 6 months and gross motor (first regression), fine motor (second regression), and cognitive (third regression) scores at 12 months, controlling for 6-month cognitive scores and neonatal status (ELGA vs. FT infants). We used raw scores in linear regression analyses because they more accurately describe the growth in outcome variables over time. Because a relevant number of children with a delay at 12 months was found only in the gross motor subscale, we performed a logistic regression to assess whether gross and fine motor standardized scores at 6 months were predictors of gross motor delay at 12 months, controlling for 6-month cognitive standardized scores and neonatal status (ELGA vs. FT infants).

## Results

The first hierarchical regression analysis showed that the 6-month gross motor score (β = 0.73, *p* < 0.001) predicted the 12-month gross motor score with a *R*^2^ of 0.53, *F*(1, 39) = 42.51, and *p* < 0.001. As presented in Table 4, when we included the 6-month fine motor score (Model 2) and neonatal status (Model 4), the *R*^2^ did not increase significantly. In contrast, when we included the 6-month cognitive score (β = 0.43, *p* = 0.023; Model 3), the *R*^2^ increased significantly to 0.59, *F*(1, 39) = 17.48, and *p* < 0.001. This suggests that the 6-month gross motor score predicted the 12-month gross motor score and the 6-month cognitive score explained an additional significant amount of variance (Δ*R^2^* = 0.06, *p* = 0.023).

**TABLE 4:** Hierarchical regression analyses models for 12-month gross motor, fine motor, and cognitive scores.

**6-month predictors**	**12-month gross motor**				**12-month fine motor**				**12-month cognitive**			
	***B***	**β**	***p***	***R*^2^**	***B***	**β**	***p***	***R*^2^**	***B***	**β**	***p***	***R*^2^**
**Model 1**												
Intercept	12.39				24.53				19.72			
Gross motor	1.06	0.73	**<0.001**	0.53	0.49	0.58	**<0.001**	0.34	0.65	0.65	**<0.001**	0.42
**Model 2**												
Intercept	11.77				0.23				21.09			
Gross motor	1.01	0.69	**<0.001**		0.37	0.44	**0.017**		0.76	0.75	**<0.001**	
Fine motor	0.08	0.05	0.743	0.53	0.20	0.21	0.233	0.36	-0.18	-0.16	0.343	0.43
**Model 3**												
Intercept	12.61				23.25				21.29			
Gross motor	0.82	0.56	**0.001**		0.31	0.37	0.058		0.71	0.71	**<0.001**	
Fine motor	–0.32	–0.20	0.271		0.08	0.09	0.699		-0.27	-0.24	0.243	
Cognitive	0.56	0.43	**0.023**	0.59	0.17	0.22	0.327	0.38	0.14	0.15	0.486	0.44
**Model 4**												
Intercept	12.49				23.42				21.71			
Gross motor	0.85	0.58	**0.001**		0.27	0.32	0.10		0.61	0.61	**0.001**	
Fine motor	–0.29	–0.18	0.317		0.05	0.05	0.825		-0.36	-0.32	0.097	
Cognitive	0.58	0.44	**0.021**		0.14	0.19	0.407		0.07	0.08	0.691	
Neonatal status	–0.81	–0.08	0.545	0.60	1.15	0.19	0.224	0.40	2.90	0.40	**0.005**	0.56

*Number of participants = 40. Raw scores of gross motor (locomotor subscale), fine motor (eye and hand coordination subscale) and cognitive skills (performance subscale) of the Griffiths Mental Development Scales were used. Neonatal status: Full term children as reference. 12-month gross motor score: Δ*R^2^* = 0.00 for Model 2 (*p* = 0.743); Δ*R^2^* = 0.06 for Model 3 (*p* = 0.023); Δ*R^2^* = 0.00 for Model 4 (*p* = 0.545). 12-month fine motor score: Δ*R^2^* = 0.03 for Model 2 (*p* = 0.233); Δ*R^2^* = 0.02 for Model 3 (*p* = 0.327); Δ*R^2^* = 0.03 for Model 4 (*p* = 0.224). 12-month cognitive score: Δ*R^2^* = 0.01 for Model 2 (*p* = 0.343); Δ*R^2^* = 0.01 for Model 3 (*p* = 0.486); and Δ*R^2^* = 0.11 for Model 4 (*p* = 0.005). Significant results (*p* < 0.05) are in bold.*

The second linear regression analysis showed that the 6-month gross motor score (β = 0.58, *p* < 0.001) predicted the 12-month fine motor score with an *R*^2^ of 0.34, *F*(1, 39) = 19.16, *p* < 0.001 (see Table 4). When we included the 6-month fine motor score (Model 2), the 6-month cognitive score (Model 3), and neonatal status (Model 4), no significant additional amount of variance was explained.

The third linear regression analysis showed that the 6-month gross motor score (β = 0.65, *p* < 0.001) predicted the 12-month cognitive score with an *R*^2^ of 0.42, *F*(1, 39) = 27.46, and *p* < 0.001. As presented in Table 4, when we included the 6-month fine motor score (Model 2) and the 6-month cognitive score (Model 3), no significant additional amount of variance was explained; whereas, when we included neonatal status (Model 4), the *R*^2^ increased significantly to 0.56, *F*(1, 39) = 10.94, and *p* < 0.001. This suggests that the 6-month gross motor score predicted the 12-month cognitive score and the neonatal status explained an additional significant amount of variance (Δ*R^2^* = 0.11, *p* = 0.005).

The logistic regression analysis showed an *R*^2^ of.307, *Wald*, *X*^2^(1) = 4.051, and *p* = 0.044, revealing that the 6-month gross motor score (B = -0.27; *OR* = 0.766) was a significant predictor of delay in gross motor development at 12 months. Our findings can be interpreted as showing that for every one unit increase in 6-month gross motor score, the odds of being delayed in the gross motor domain at 12 months decreased by 0.27 unit.

## Discussion

This study showed that early gross motor abilities have intra-domain cascading effects on motor development and cross-domain cascading effects on cognitive development and can be considered an early index for identifying delays in the gross motor domain. Specifically, 6-month gross motor skills were related to 12-month gross motor, fine motor, and cognitive skills and predicted 12-month gross motor delays. Six-month cognitive skills explained an additional amount of variance of 12-month gross motor skills, whereas neonatal status explained an additional amount of variance of 12-month cognitive skills.

Our study provides new evidence on the intradomain relationships between early and later motor development in ELGA infants. In particular, our results revealed that gross motor skills at 6 months predicted gross motor skills at 12 months and played a crucial role in predicting fine motor skills at 12 months. As argued by previous studies, gross motor skills and, particularly, the achievement of adequate postural sitting and head control promote development of arm and hand function (Plantiga et al., 1997), grasping and visuo-motor integration (Wang et al., 2011), and reaching (Rochat and Goubet, 1995; Carvalho et al., 2008) as well as the quantity and quality of object exploratory behaviors (Soska and Adolph, 2014; Marcinowski et al., 2019). In accordance with these arguments, our findings suggest that the accomplishment of adequate gross motor development is a relevant prerequisite for later perceptual motor integration, object manipulation, reaching, grasping, and functional hand skills. Therefore, an uneven development of gross motor abilities may have cascading effects not only on later gross motor functions but also on fine motor ones. Interestingly, we also found that cognitive skills at 6 months explained an additional portion of variance in gross motor development at 12 months. This suggests that in addition to early gross motor skills, cognitive skills, more than the neonatal condition, may affect later gross motor outcomes.

Our findings revealed cross-domain cascading effects of early gross motor skills on 12-month cognitive skills. This result provides new evidence in extremely preterm infants, showing an association between early gross motor skills, assessed at 6 months, and cognitive skills already evident by 12 months. A previous study (Lefebvre et al., 2016) reported this association with cognitive skills at 18 months. Our results showed also that the relationship between early gross motor skills and later cognitive skills is present not only when considering specific gross motor skills (i.e., the quality of postural control or of general movements), as found in previous studies on the preterm population (Wijnroks and van Veldhoven, 2003; Oudgenoeg-Paz et al., 2017), but also when considering global gross motor skills assessed with a standardized tool. This confirms that motor development is a driving force for development in other domains, such as the cognitive one. Indeed, the acquisition of early motor skills and, in particular, of gross motor skills, provides infants with new learning opportunities, initiating, in this way, developmental cascades that affect subsequent cognitive achievements. For example, as shown in Wijnroks and van Veldhoven (2003) study, preterm infants with better postural control at 6 months scored higher in cognitive measures 6 and 18 months later. Indeed, the acquisition of good postural control, and, in particular, sitting without support, is a necessary prerequisite for goal-oriented behaviors, such as reaching (Rochat and Goubet, 1995). Reaching objects, in turn, enables infants to explore them and learning their characteristics, contributing in this way to infant cognitive development (Ruff et al., 1984; Zuccarini et al., 2017). Evidence that motor and cognitive development are intertwined has been provided by a growing body of theories (Smith, 2005) and related empirical behavioral and neuropsychological studies (Oudgenoeg-Paz et al., 2017). Indeed, neuroimaging studies have shown that several cognitive and motor tasks require the activation of the same neural areas (e.g., the dorsolateral prefrontal cortex and cerebellum), suggesting that motor and cognitive skills may involve the same underlying neural system and need to be studied not in insolation (Diamond, 2000).

Besides early gross motor skills, we have demonstrated that neonatal status explained an additional portion of the variance of cognitive development in the first year of life. This finding brings new evidence that preterm birth contributes to determining atypical developmental trajectories (Sansavini et al., 2011), with cascading effects on general cognitive development, as found in previous studies (Sansavini et al., 2011, 2014). To deeply understand these effects, future studies should examine in the preterm population the impact of early motor skills on specific cognitive functions emerging at the end of the first year, such as the beginning of cognitive planning, inhibition, and selective attention (Downes et al., 2018).

We also found correlations between fine motor and cognitive scores at 6 months and fine motor and cognitive scores at 12 months. However, when we considered gross motor skills in the regression model and controlled for cognitive skills at 6 months and neonatal status, the variance of 12-month fine motor and cognitive scores was explained mostly by the 6-month gross motor scores. This result may have several explanations. On one hand, as the neuroconstructivist approach assumes, developmental competencies are highly interrelated in the first year of life, whereas they become more differentiated and specialized in the following years (Karmiloff-Smith, 2009). Thus, it seems that gross motor skills are related to and significantly affect fine motor and cognitive skills during the first year of life. On the other hand, motor development proceeds from the proximal parts of the body, i.e., head and trunk, and the proximal functions, i.e., the postural control, to the distal parts of the body, i.e., hands and feet, and distal functions, i.e., fine motor skills (Case-Smith et al., 1989). According to this model, a study on preterm infants (Wang et al., 2011) showed that a high percentage of variance of fine motor skills was explained by the scores of postural control at 6 months, suggesting that there is a functional relationship between proximal motor control and the development of distal functions. Furthermore, as mentioned above, the ability to reach objects in the environment, allowing to explore them, is considered a relevant precursor of later cognitive development. Taking into account this evidence, we can speculate that, although, fine motor and cognitive scores at 6 months correlated with fine motor and cognitive scores at 12 months, the main predictive power was coming from 6-month gross motor skills that appear as the foundation for the other two domains.

Our findings also showed that gross motor scores at 6 months predicted delays in gross motor development at 12 months. This result confirms and expands previous findings by Lefebvre et al. (2016) showing that gross motor skills in the first year of life play a crucial role in predicting later motor outcomes, even in ELGA infants without neurological damage. Importantly, this result is also in line with the recommendations of the American Academy of Pediatrics for detecting infants at risk of motor delays. Indeed, at the recommended screening visit at 9 months of age, specific gross motor skills, such as sitting well without support or rolling to both sides, should have been acquired, and their absence at that age is an index of delay (Noritz et al., 2013). Along these lines, at 12 months, the inability to stand with support or a still immature trunk control may suggest a motor insufficiency (Pin et al., 2010). Indeed, in our sample, 25% of the ELGA infants presented a gross motor delay, characterized by the inability to stand with support or alone and begin to walk. A delay was also identified in infants born at term, but only in 10% of the 12-month FT group. Pin et al. (2010) also found a high variability in gross motor skills at 12 months, that tended to decrease at later ages. As Mansson and Stjernqvist (2014) showed, the prevalence of moderate–severe delays in the gross motor domain in extremely preterm infants decreased around 30 months of age. Further studies should thus investigate the persistence of delay in gross motor development among healthy extremely preterm children after the first year of life. Indeed, a longitudinal motor assessment is highly recommended to increase the predictive and discriminative value of the assessment as well as to detect, across the first year, infants at risk for later motor disorders (Spittle et al., 2008).

### Limitations and Future Directions

Some limitations of the current study need to be taken into account. First, we based our assessment of gross and fine motor development on a standardized test, the GMDS-R, commonly used in clinical practice and scientific studies conducted with the preterm population (Sansavini et al., 2011, 2014; Mansson and Stjernqvist, 2014), and recognized as a valid tool for examining motor development in the first year of life (Evensen et al., 2009; Greene et al., 2012). However, other tools developed to evaluate specific gross motor behaviors—for example, spontaneous general movements and postural development- and specific fine motor behaviors- for example, reaching kinematics or object exploration—should also be used in future studies to deeply investigate the relationships between gross and fine motor skills and later developmental outcomes. Indeed, as other studies on typically developing and ELGA infants have suggested (Charitou et al., 2010; Pin et al., 2010), researchers in the future could examine the achievement of specific gross motor milestones, for example, unsupported sitting, fine motor milestones, proficient planning, control of reaching, or complex object exploratory patterns (Fallang et al., 2005; Zuccarini et al., 2016, 2017; Kaul et al., 2019), as well as, their relationships with spontaneous general movements, e.g., anti-gravity limbs movements, showed by preterm infants in the very first months of life (Miyagishima et al., 2018). This could be helpful for more deeply understanding intradomain and cross-domain relationships and for identifying which specific early gross and fine motor skills predict later motor and cognitive development in ELGA infants.

A second limitation is the small sample size of infants classified as delayed in the fine motor and cognitive domains at 12 months, hence we could not address the question of antecedents and predictors of delays in an exhaustive way. A larger sample of extremely preterm infants should thus be recruited in future studies to detect a sufficient number of infants showing motor and cognitive delays by the end of the first year of life.

Third, we focused on relationships between early motor skills and motor and cognitive development in the first year of life. Indeed, at 6 and 12 months we assessed cognitive skills in terms of performance abilities that can be precursors of higher level cognitive functions, i.e., problem solving or executive functions, often impaired in preterm infants, that develop later than the first year of age (Oudgenoeg-Paz et al., 2017). Thus, it would be important in future studies to examine these relationships assessing higher level cognitive functions at later ages. Furthermore, some studies have demonstrated that early motor skills are also strictly linked to other developmental domains, for example, the language domain, in typically developing infants (Iverson, 2010), and in ELGA infants (Zuccarini et al., 2017, 2018), and several psychological functions, e.g., perception, spatial cognition, social, and emotional development, in typically developing infants (Campos et al., 2000). One noteworthy observation is the relationships between early motor skills and other domains seem to become weaker at preschool and school age in typically developing children (Libertus and Hauf, 2017). Therefore, in future studies, researchers should examine the persistence of these relationships in the ELGA population as a function of age, taking into account associations with other domains.

Fourth, our study included healthy extremely preterm infants compared to FT infants. Researchers in future studies could examine a sample of preterm infants with a wider range of gestational ages to analyze whether the same intra-domain and cross-domain cascading effects are present. For example, Kaul et al. (2019) found that the associations between early reaching and later neurodevelopment changed as a function of gestational age; specifically, among extremely preterm infants, visuomotor planning and control of reaching were strongly related to later cognition, whereas among very preterm infants, executional aspects of reaching (i.e., bimanual coupled reaches) were more strongly related to later cognitive development. Moreover, in the future, increasing the sample size of preterm as well as of full-term infants would allow to explore whether these associations differ in function of neonatal status (ELGA vs. FT).

Finally, in the current study, we did not consider the environmental influences on infant’s motor performance such as infant’s early motor experiences. In the future, those aspects could be considered to further investigate intradomain and cross-domain relationships in ELGA and FT infants in the first years of life (Spittle et al., 2008).

### Conclusions and Clinical Implications

In sum, our findings highlighted that gross motor skills at 6 months have cascading effects on motor and cognitive development at 12 months. Furthermore, gross motor skills at 6 months appear a reliable index for identifying later delays in the gross motor domain.

The results of this study have clinical implications for follow-up and intervention programs designed for ELGA infants. Considering the crucial role that early motor skills and, in particular, early gross motor skills play in later motor and cognitive development across the first year of life, assessing and supporting these abilities as soon as possible appear highly relevant. In particular, our findings underscore the relevance for clinicians to assess gross motor skills at 6 months and identifying early gross motor behaviors that could have an impact on later development. For example, as shown in a previous study on preterm infants, difficulties in postural control, i.e., a less stable control of the trunk, extension of the elbows, or signs of hyperextension, at 6 months, have a significant impact on later cognitive tasks, such as the ability of problem solving (Wijnroks and van Veldhoven, 2003). Another study on infants at high familiar risk for Autism Spectrum Disorders (LeBarton and Iverson, 2016) has shown that the acquisition of stability in sitting at around 6 months has cascading effects on later communicative development. Therefore, detecting motor delay at an early stage, and, in particular, motor delays in gross motor skills, and implementing effective interventions could reduce the impact of impaired early motor skills on later development, both within the motor domain, and across other domains (Spittle et al., 2008). As underscored recently, interventions on preterm infants should be carried out beyond the first months of life and should include guidelines for parents and caregivers on motor development (Valentini et al., 2019). Along these lines, a meta-analysis by Spittle et al. (2012) suggested that interventions focusing on preterm infants (e.g., physiotherapy), and their parents (e.g., parent–infant relationships) had a positive impact on motor development and cognitive development, improving outcomes in these domains during infancy and preschool age. Consistently with these findings, a recent randomized trial study confirmed that improving parenting practices, for example, guiding parents to teach their infants new skills, such as postural control or grasping toys, had a significant impact especially on preterm infants’ motor skills (Colditz et al., 2019). In conclusion, our findings highlight the relevance of early motor skills to preterm infants’ development, and thus point to the importance of assessing those skills and implementing early interventions, which also involve the caregivers.

## Data Availability Statement

The datasets generated for this study are available on request to the corresponding author.

## Ethics Statement

All study procedures met the ethical guidelines for protection of human participants, including adherence to the legal requirements of the country, and received a formal approval by the Ethical Committee of the Hospital of the University of Bologna. The parents of all infants provided written informed consent for participation in the study, data analysis, and anonymized data publication.

## Author Contributions

AS and AG designed and conceptualized the study, methods, data collection, and analyses. GF supervised the medical data collection of the preterm sample and medical aspects of the method. SS collected the data. MZ, AG, and AS analyzed the data, drafted, revised, and reviewed the manuscript. AS supervised the study. All authors approved the final manuscript.

## Conflict of Interest

The authors declare that the research was conducted in the absence of any commercial or financial relationships that could be construed as a potential conflict of interest.
