# Supplementary material for: Do 6-Month Motor Skills Have Cascading Effects on 12-Month Motor and Cognitive Development in Extremely Preterm and Full-Term Infants?
Source: Front Psychol. 2020 Jun 26;11:1297. doi: 10.3389/fpsyg.2020.01297 (PMC7332837; doi:10.3389/fpsyg.2020.01297)
Supplement: Supplementary file 1 [file Table_1.pdf]

Supplementary File 1. Pearson's correlations among gross motor, fine motor and cognitive scores at 6 months.

	6-months					
	**Gross motor**		**Fine motor**		**Cognitive**	
	r	***p***	***r***	***p***	***r***	***p***
**6-months**						
Gross motor	-	**-**	.661	**<.001**	.687	**<.001**
Fine motor	-	**-**	-	**-**	.776	**<.001**

Note. Raw scores of gross motor (locomotor subscale), fine motor (eye and hand coordination subscale) and cognitive skills (performance subscale) of the Griffiths Mental Development Scales were used.
Significant results ($p < .05$) are in bold.
