# Supplementary material for: Do 6-Month Motor Skills Have Cascading Effects on 12-Month Motor and Cognitive Development in Extremely Preterm and Full-Term Infants?
Source: Front Psychol. 2020 Jun 26;11:1297. doi: 10.3389/fpsyg.2020.01297 (PMC7332837; doi:10.3389/fpsyg.2020.01297)
Supplement: Supplementary file 2 [file Table_2.pdf]

Supplementary File 2. Pearson's correlations among gross motor, fine motor and cognitive scores at 12 months.

	**12 months**					
	**Gross motor**		**Fine motor**		**Cognitive**	
	r	*p*	*r*	*p*	*r*	*p*
**12-months**						
Gross motor	-	**-**	.478	**.002**	.486	**.001**
Fine motor	-	**-**	-	**-**	.507	**.001**

Note. Raw scores of locomotor (locomotor subscale), eye and hand coordination (eye and hand coordination subscale) and performance (performance subscale) of the Griffiths Mental Development Scales were used.
Significant results ($p < .05$) are in bold.
